# GlcNAc6ST3 is a keratan sulfate sulfotransferase for the protein-tyrosine phosphatase PTPRZ in the adult brain

**DOI:** 10.1038/s41598-019-40901-2

**Published:** 2019-03-13

**Authors:** Narentuya, Yoshiko Takeda-Uchimura, Tahmina Foyez, Zui Zhang, Tomoya O. Akama, Hirokazu Yagi, Koichi Kato, Yukio Komatsu, Kenji Kadomatsu, Kenji Uchimura

**Affiliations:** 1 Department of Biochemistry, Nagoya University Graduate School of Medicine, Nagoya, 466-8550 Japan; 2 Department of Neuroscience, Research Institute of Environmental Medicine, Nagoya University, Nagoya, 464-8601 Japan; 3 Department of Pharmacology, Kansai Medical University, Osaka, 570-8506 Japan; 4 Nagoya City University Graduate School of Pharmaceutical Sciences, Nagoya, 467-8603 Japan; 5 Institute for Molecular Science and Okazaki Institute for Integrative Bioscience, Okazaki, 444-8787 Japan; 6 National Institute for Physiological Sciences, National Institutes of Natural Sciences, Okazaki, 444-8585 Japan; 7 Unité de Glycobiologie Structurale et Fonctionnelle, UMR 8576 CNRS, Université de Lille, 59655 Villeneuve d’Ascq, France; 8 Present Address: Department of Pharmaceutical Sciences, North South University, Dhaka-1229 Bashundhara, Bangladesh

## Abstract

Keratan sulfate (KS) is a carbohydrate side chain covalently attached to extracellular proteoglycans. KS is composed of disaccharide units of 6-sulfated *N*-acetylglucosamine (GlcNAc) and galactose. We have previously shown that GlcNAc-6-*O*-sulfotransferase (GlcNAc6ST) 1 encoded by *Chst2* is an enzyme necessary for the synthesis of GlcNAc-6-sulfated KS chains that are required for neuronal plasticity in the visual cortex of the mouse brain during the critical period, but not in adulthood. Here, we show that GlcNAc-6-sulfated KS recognized by the R-10G anti-KS antibody, of which the minimum epitope structure is Galß1-4GlcNAc(6S)ß1-3Galß1-4GlcNAc(6S), distributes diffusely in neuropils and presents densely in close proximity to the perineuronal region of the perineuronal net (PNN)-positive neurons in the adult visual cortex. Surprisingly, GlcNAc6ST3, which was discovered as an intestinal GlcNAc6ST encoded by *Chst5*, is a major brain KS sulfotransferase expressed in oligodendrocytes in adulthood. Moreover, we identified an isoform of the protein-tyrosine phosphatase PTPRZ as a R-10G-reactive KS proteoglycan. These results indicate that GlcNAc6ST3 may play a role in synthesis of a component of PNN in the adult brain, and that the KS-modified isoform of PTPRZ encoded by *Ptprz1* could be an extracellular molecule associated with PNNs.

## Introduction

Extracellular matrix molecules, such as chondroitin sulfate proteoglycans (CSPGs) and tenascins, interact with each other to fill the extracellular spaces and interpose among neurons and glia in the brain^1–4^. CSPGs are also present on neuronal cell surfaces^5,6^, forming networks known as perineuronal nets (PNNs)^7,8^. PNNs are heterogeneous in composition^9^, and their formation is concurrent with the termination of the critical period^7,10,11^. We have previously shown that CSPG phosphacan, an isoform of protein-tyrosine phosphatase receptor type Z (PTPRZ) modified with keratan sulfate (KS), is distributed diffusely in the extracellular space and is required for cortical plasticity during the critical period^12^. In the adult brain, however, distribution of the KS and their proteoglycan core proteins remain largely elusive.

KS is a glycosaminoglycan side chain, consisting of repeating mono- or di-sulfated disaccharides of *N*-acetylglucosamine (GlcNAc) and galactose (Gal)^13,14^. In the brain, KS chains attach covalently to a core protein mainly through *O*-mannose-linked glycans in its rather short forms^15–17^. Sulfated motifs within KS can be differentially detected with specific antibodies. The R-10G monoclonal antibody recognizes KS structures containing mono-sulfated disaccharides of 6-sulfated GlcNAc and Gal^18^. The minimum epitope structure is Galß1-4GlcNAc(6S)ß1-3Galß1-4GlcNAc(6S)^19^. The 5D4 monoclonal antibody detects KS structures composed of di-sulfated disaccharides of GlcNAc and Gal, both 6-sulfated. The Golgi-resident sulfotransferases catalyze C-6 sulfation modifications of GlcNAc and Gal residues of KS^14^. *N*-Acetylglucosamine 6-*O*-sulfortansferase (GlcNAc6ST) transfers a sulfate to GlcNAc. Five and four members of the GlcNAc6ST family have been identified in humans and mice, respectively^20^. Heparan sulfate 6-*O*-sulfotransferases that transfer sulfate at C-6 of the *N*-sulfoglucosamines are distinct from the members of the GlcNAc6ST family^21^. We have previously generated a GlcNAc6ST1-deficient mouse model^22^ and reported that deficiency in the GlcNAc6ST1 gene, *Chst2*, led to a partial reduction of the GlcNAc-6-sulfated KS of phosphacan/PTPRZ in the early postnatal brain^23^ and in the critical period^12^. On the other hand, the GlcNAc-6-sulfated KS content was not changed in the adult stage by the deletion of the GlcNAc6ST1 gene^12^. In order to determine whether other members of the GlcNAc6ST family act on the KS in the adult brain, we analyzed mice deficient in either GlcNAc6ST2 encoded by *Chst4*^24^, or GlcNAc6ST3, which was discovered initially as an intestinal GlcNAc6ST^25^ and is encoded by *Chst5*^26^. Disrupting *Chst7* resulted in the GlcNAc6ST4-deficient mice that were analyzed as well. Surprisingly, we found that disruption of GlcNAc6ST3, an intestinal enzyme, eliminated almost all GlcNAc-6-sulfated KS recognized by the R-10G anti-KS antibody in the adult brain, and that GlcNAc6ST3 was selectively expressed in oligodendrocyte precursor cells (OPCs) and the newly formed oligodendrocytes in the adult brain. Moreover, we identified phosphacan/PTPRZ as a major R-10G-positive KS-modified CSPG in the adult brain. The R-10G-positive KS-modified phosphacan/PTPRZ exists diffusely within neuropils and densely in close proximity to perineuronal regions of a subset of PNN-positive neurons in the adult brain cerebral cortex. These results indicate that GlcNAc6ST3 in oligodendrocytes is a major KS enzyme in the adult brain, and that GlcNAc6ST3 may play a role in synthesis of a PNN component, and the KS-modified isoform of PTPRZ could be associated with PNNs.

## Results and Discussion

### The R-10G-reactive GlcNAc-6-sulfate KS is born on a CSPG in the adult mouse brain

**Figure 1:**
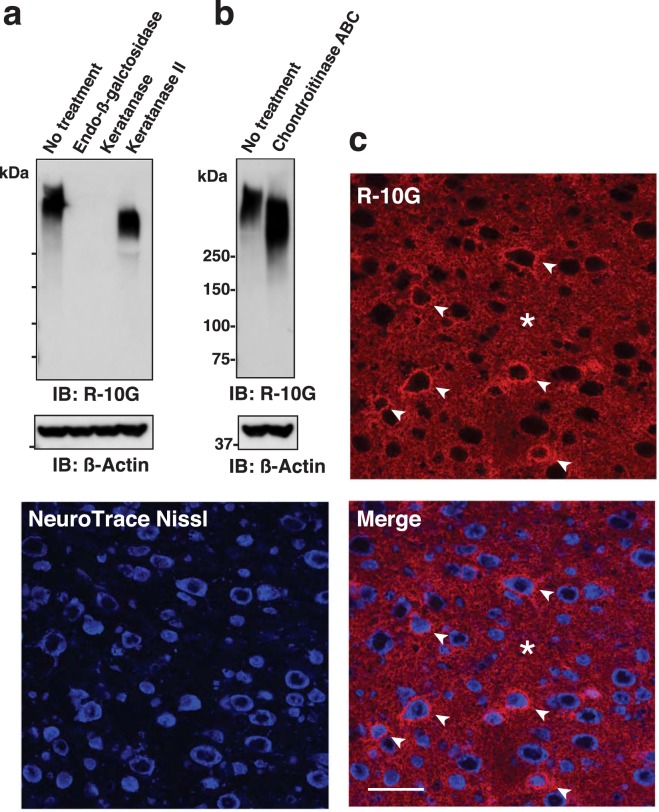
Expression and localization of R-10G-reactive keratan sulfate/chondroitin sulfate proteoglycans in the cerebral cortex of adult mice. (**a**,**b**) R-10G monoclonal antibody recognizes GlcNAc-6-sulfated keratan sulfate (KS)^18,19^. Expression of the R-10G KS epitope in the 1% Triton-soluble fractions prepared from the cerebral cortex in adult wild-type (WT) mice is shown with or without pretreatments with KS-degrading enzymes (**a**) or chondroitinase ABC. (**b**) R-10G-reactive band signals were eliminated by endo-ß-galactosidase or keratanase pretreatment. ß-Actin was used as a loading control. (**c**) Brain sections from adult WT mice were immunostained with R-10G (*red*) followed by NeuroTrace Nissl staining (*blue*). The NeuroTrace blue-fluorescent Nissl stain was used to visualize neurons. A representative confocal microscope image of the cerebral cortex is shown (n = 3). R-10G staining signals in pericellular (*arrowheads*) and intercellular spaces (*asterisk*) were detected. Full-length blot images are presented in Supplementary Fig. S8. Scale bar: 20 µm.

**Figure 2:**
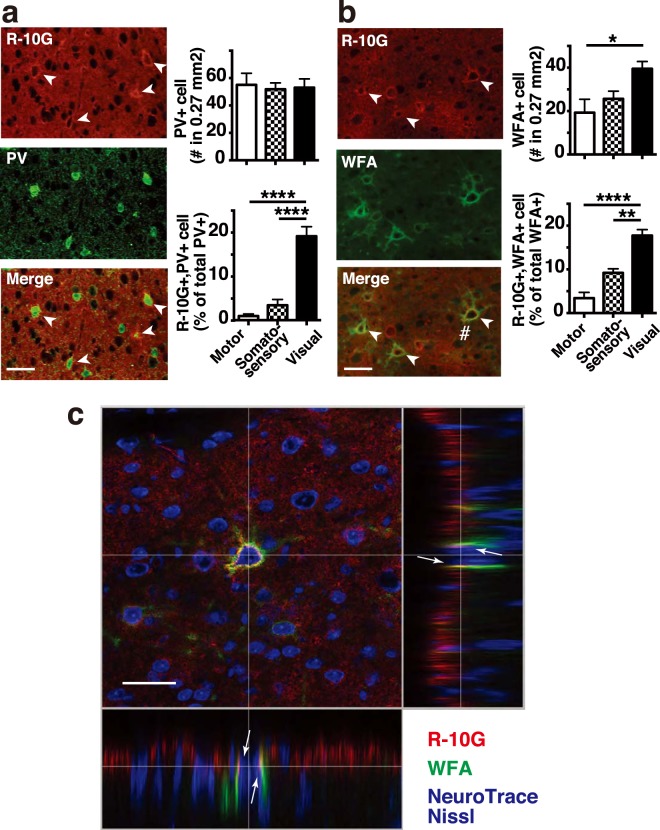
Pericellular localization of R-10G-reactive KS epitopes in perineuronal net-positive neurons in the visual cortex of adult mice. (**a**) Brain sections from adult mice were immunostained with R-10G (*red*) and an anti-parvalbumin (PV) antibody (*green*). Representative fluorescence microscopic images of the visual cortex are shown (n = 3). R-10G-reactive KS is found pericellularly in the PV-positive neurons (*arrowheads*). The graphs show the number of PV-positive cells, and the percentage of R-10G-positive cells compared to the total number of PV-positive cells in motor, somatosensory, and visual cortices. The numbers of PV-positive cells and R-10G-positive cells were counted and quantitated in each cortex (n = 3). Digital images of five to six randomly selected fields in each cortex were captured. A subset of R-10G-positive/PV-positive neurons was preferentially detected in the visual cortex. (**b**), Adult mouse brain sections were immunostained with R-10G (*red*) and a FITC-labeled WFA lectin (*green*). Representative fluorescence microscopic images in the visual cortex are shown (n = 3). R-10G-reactive KS is found pericellularly in WFA-positive neurons (*arrowheads*). The graphs show the number of WFA-positive cells, and the percentage of R-10G-positive cells compared to the total number of WFA-positive cells in motor, somatosensory, and visual cortices. The number of WFA-positive cells and R-10G-positive cells were counted and quantitated in each cortex (n = 3). Digital images of three to eight randomly selected fields in each cortex were captured. Data are means ± SEM. *P < 0.05, **P < 0.01, ****P < 0.0001. (**c**) A confocal Z-stack high magnification image of an area indicated with *#* in (**b**) is shown. Pericellular R-10G signals (*red*) in close proximity to the NeuroTrace Nissl-stained soma (*blue*) of a WFA (*green*)-positive neuron are indicated (*arrows*). Scale bars: 20 µm.

We previously showed that no or minimal expression of KS epitopes recognized by the 5D4 antibody was observed in the adult mouse brain^23^, while GlcNAc-6-monosulfated KS, which is recognized by the R-10G antibody, was expressed at the level comparable to that in the critical period brain^12^. To confirm that the R-10G recognized molecule is indeed KS-modified, we pretreated the brain samples of adult wild-type (WT) mice with KS-degrading enzymes. Pre-digestion with endo-ß galactosidase and keratanase, which hydrolyze ß-galactosidic linkages in KS chains composed of non-sulfated Gal and 6-sulfated GlcNAc disaccharides (Galß1-4GlcNAc(6S)), eliminated the R-10G-reactive KS (Fig. 1a). The cleavage of the ß-galactosidic bond by keratanase requires C-6 sulfate modification of the adjacent GlcNAc residue. These data and the recent report that the R-10G antibody does not recognize *N*-acetyllactosamines (Galß1-4GlcNAc)^19^ indicate that the R-10G-positive band is indeed a GlcNAc-6-sulfated KS-modified molecule. Keratanase II hydrolyses 1,3-ß-glucosaminidic linkages to Gal residues in KS composed of Galß1-4GlcNAc(6S) and Gal-6- and GlcNAc-6-sulfated disaccharides (Gal(6S)ß1-4GlcNAc(6S)). Band intensity was not altered but the apparent molecular weight of the R-10G reactive band at MW > 500 kDa shifted slightly downwards after treatment with keratanase II (Fig. 1a). This R-10G-reactive protein might also be modified with a distinct KS chain, which is composed of Gal(6S)ß1-4GlcNAc(6S) disaccharides non-immunoreactive with the R-10G antibody. It is also plausible that both the keratanase II-susceptible structure and the keratanase-susceptible R-10G epitope present in a single KS chain, of which the former structure exists only at the non-reducing terminus. Sialic acid and fucose modifications within the KS may give rise to inhibition of the keratanase II activity to the Galß1-4GlcNAc(6S) disaccharides as previously described^27^. A band shift with a higher potency than that seen in keratanase II treatment was observed after chondroitinase ABC treatment (Fig. 1b). Specifically, the band shifted down to approximately 320 kDa after chondroitinase ABC treatment. The susceptibility to chondroitinase ABC indicated that the R-10G-reactive protein is modified with chondroitin sulfate chains. These results indicated that the R-10G-reactive protein is a CSPG modified with GlcNAc-6-sulfated KS. To investigate the cellular localization of the KS/CSPG, we performed immunohistochemical staining with R-10G on cryostat-cut brain sections prepared from adult WT mice. R-10G staining signals were punctate and seen diffusely in neuropils/extracellular spaces within the whole area of the cerebral cortices (Fig. 1c). Staining for synaptophysin (SYP), a pan-presynaptic marker, and GLAST, an astrocyte plasmalemmal marker, was not colocalized with this diffuse R-10G staining (Fig. S1). PNNs selectively surround inhibitory interneurons that express parvalbumin (PV), and are labeled with a well-established marker, *Wisteria floribunda* agglutinin (WFA)^8^. Intriguingly, dense R-10G staining in the pericellular regions was observed (Fig. 1c) in a subset of neurons that are PV-positive or WFA-positive within the cerebral cortex (Fig. 2a,b). These pericellular signals were subtle in a PV-positive cell subset within motor and somatosensory cortices (1% and 3% of total PV-positive cells, respectively) (Fig. 2a). In the visual cortex, the pericellular signals were seen in 20% of total PV-positive cells (Fig. 2a). Similarly, these signals are less prevalent in the WFA-positive cell subset within motor and somatosensory cortices (3% and 9% of WFA-positive cells, respectively) than in the visual cortex, where 18% of WFA-positive cells were R-10G-positive (Fig. 2b). Confocal microscopy analysis showed that some of the pericellular R-10G signals exist densely in close proximity to perineuronal regions (Figs 2c and S2). These results strongly indicate that R-10G reactive KS/CSPGs are accumulated in a subset of inhibitory intercortical neurons in the adult brain cortex with the preferential localization in the visual cortex. These neurons may include subsets of the R-10G positive neurons seen in the critical period^12^.

### GlcNAc6ST3 is a major sulfotransferase for the R-10G-positive KS/CSPG in the adult mouse brain

**Figure 3:**
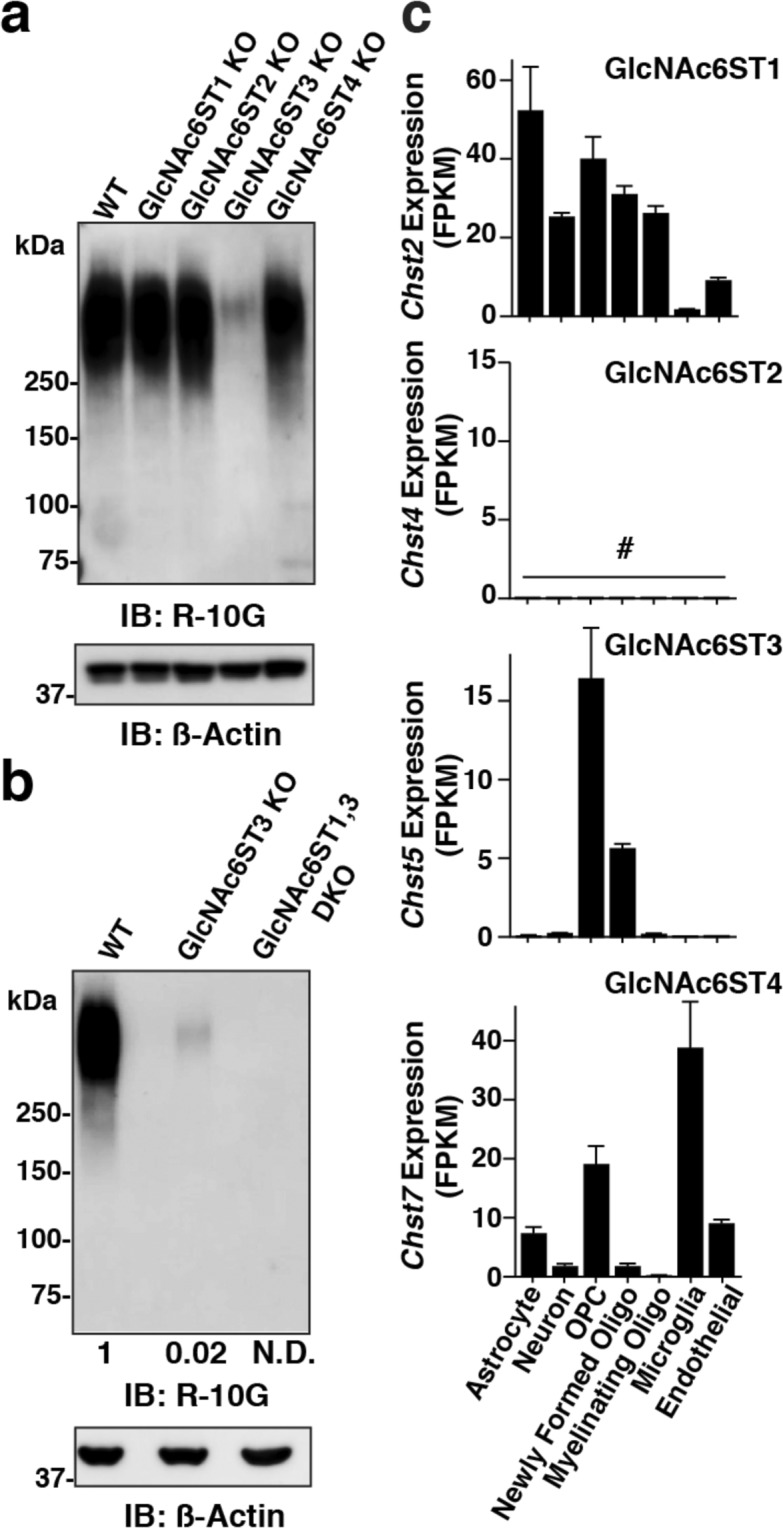
GlcNAc6ST3 in oligodendrocytes is a major sulfotransferase of R-10G-reactive KS in the cerebral cortex of adult mice. (**a**,**b**) Expression of the R-10G KS epitope in the 1% Triton-soluble fractions prepared from the cerebral cortex of adult WT mice and GlcNAc6ST1, 2, 3, and 4 single KO, and GlcNAc6ST1, 3 doubly-deficient (DKO) mice. The samples were pretreated with the chondroitinase ABC before western blotting. Representative results are shown (n = 3 in **a**, n = 2 in **b**). Densitometric quantitative analysis was used to measure the intensities of the bands in b (*open arrowhead*). The numbers denote average values relative to WT. N.D., not detected. ß-Actin was used as a loading control. (**c**) RNA-seq transcriptome results of the genes of each GlcNAc6ST family member in indicated cell types are shown. Cells were prepared from the cerebral cortex of adult mice^49^. Columns with mean values lesser than the threshold value are indicated by *#*^49^. Full-length blot images are presented in Supplementary Fig. S8.

We have previously shown that GlcNAc6ST1 is a sulfotransferase for the R-10G positive KS/CSPG in the mouse brain during the critical period^12^. However, deletion of GlcNAc6ST1 showed a level of the R-10G-positive KS comparable to that of the WT control in the adult brain. To identify which GlcNAc6ST is responsible for the synthesis of the cerebral R-10G-positive KS in the adult brain, we employed GlcNAc6ST1-KO, GlcNAc6ST2-KO, and GlcNAc6ST3-KO mice^22,26^. We newly generated GlcNAc6ST4-KO mice (Fig. S3), which showed no apparent gross abnormality. Surprisingly, the GlcNAc6ST3-KO mouse brain showed dramatically reduced levels of the R-10G-positive KS, while others showed levels equivalent to those of WT mice (Fig. 3a). The dense pericellular R-10G signals and the diffuse signals in neuropils were negligible in the visual cortex of the adult GlcNAc6ST-3 KO mice, while other KO mice showed the R-10G signals comparable to those in WT mice (Fig. S4). The low level (~2% of the WT) of the R-10G immunoreactivity seen in the GlcNAc6ST3-KO cortex was abolished in GlcNAc6ST1 and GlcNAc6ST3 doubly-deficient (DKO) mice (Fig. 3b), indicating that GlcNAc6ST3 is a major sulfotransferase for the KS/CSPG in adulthood, and that GlcNAc6ST1 merely contributes to the GlcNAc-6-sulfation of KS in the adult mouse brain. The trace amount of the diminishment did not reach a significant level in our previous work^12^. GlcNAc6ST1-KO mice did not show altered ocular dominance plasticity in the adult mouse brain^12^. It is unlikely that the low level of the GlcNAc6ST1-dependent sulfation on the KS/CSPG is involved in the ocular dominance plasticity in adulthood. Substrates for GlcNAc6ST1 in the adult brain are unknown. We found that 6-sulfations of GlcNAc residues that are ß1-2-linked to α1-3-branched mannose residues within *N*-glycans are abolished in adult GlcNAc6ST1-KO mouse brains using a multidimensional HPLC method^28^(Figs S5 and S6). It is likely that a major substrate of GlcNAc6ST1 in adult brains is an *N*-glycan rather than KS. Results of the *in vitro* sulfotransferase assay also support this possibility^29^. The GlcNAc6ST1 activity is related to pathological conditions in adult brains, as previously described^27,30^. Examining ocular dominance plasticity in GlcNAc6ST3-KO and GlcNAc6ST1, 3 DKO adult mice will address the question if GlcNAc-6-sulfation on the R-10G reactive KS/CSPG contributes to experience-dependent changes in the visual responses of cortical neurons in the adult brain. The ocular dominance shift resulting from monocular deprivation by recording visual evoked potentials from the binocular region of the visual cortex will be assessed in these KO mice^12^.We then tested which cell types express the members of the GlcNAc6ST family. mRNA expression of the GlcNAc6ST1 gene, *Chst2*, was seen broadly at a low level in microglia (Fig. 3c). Expression of the GlcNAc6ST2 gene, *Chst4*, was below the threshold of detection in either cell type of the adult brain. Interestingly, mRNA of the GlcNAc6ST3 gene, *Chst5*, was selectively expressed in OPCs and newly formed oligodendrocytes (Fig. 3c). Expression of the GlcNAc6ST4 gene, *Chst7*, was observed in astrocytes, OPCs, microglia, and endothelial cells in the brain (Fig. 3c). These results indicate that the major source of the R-10G-reactive KS/CSPG in neuropils and in the vicinity of the subsets of PV-positive and WFA-positive neurons is oligodendrocytes in the adult brain. GlcNAc6ST3 was previously identified as an intestinal GlcNAc6ST^25^. Common regulatory mechanism of the mRNA expression in oligodendrocytes and the intestine is an interesting topic. A possible link between the R-10G reactive KS/CSPG in oligodendrocytes and neuronal functions could be the subject of a future investigation. R-10G is a marker antibody for human-induced pluripotent stem cells^18^. Identification of the GlcNAc6ST responsible for the synthesis of the R-10G epitope in these cells is another future topic.

### Phosphacan/PTPRZ is a major R-10G-positive KS/CSPG in the adult mouse brain

**Figure 4:**
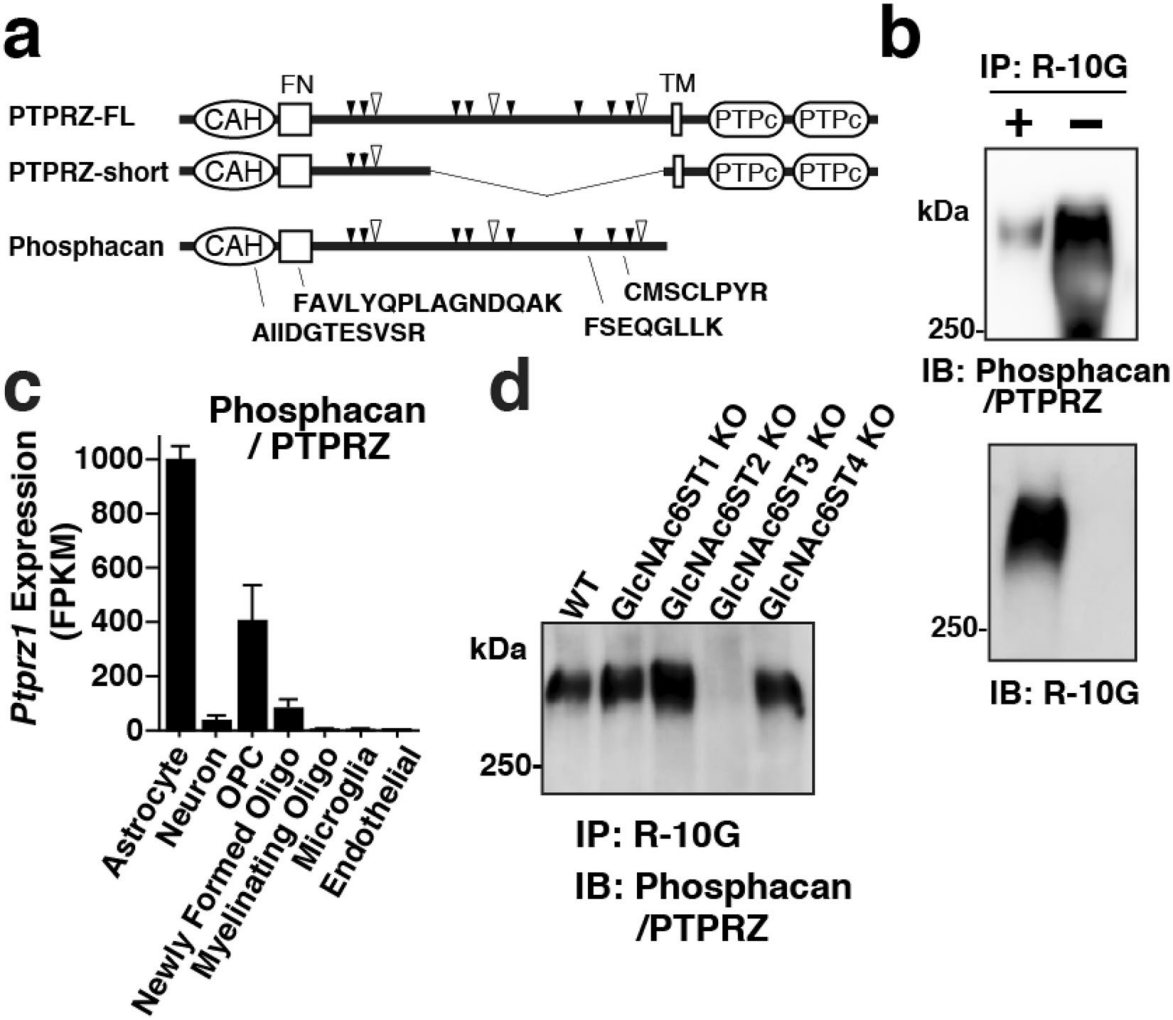
Phosphacan/PTPRZ is identified as a GlcNAc6ST3-modifying KS/CSPG expressed in the cerebral cortex of adult mice. (**a**) R-10G immunoprecipitated proteins (MW > 300 kDa) prepared from the cerebral cortex of adult WT, GlcNAc6ST2-KO, and GlcNAc6ST3-KO mice were subjected to an LC-MS/MS analysis (Fig. S7). Positions and sequences of the four identified peptides in the samples of WT and GlcNAc6ST2-KO mice, but not GlcNAc6ST3-KO mice, corresponding to phosphacan/PTPRZ are indicated. Three major isoforms of phosphacan/PTPRZ expressed in the brain are depicted. Positions of the CS GAG modification sites (*open arrowheads*) and those of the *O*-mannose modifications (*closed arrowheads*) are indicated^50,51^. The short splicing isoform lacks the peptide stretch of the PTPRZ full-length (PTPRZ-FL), which contains the sites of CS and *O*-mannose modifications. Phosphacan is the secreted isoform of PTPRZ lacking transmembrane domain (TM) and the dual phosphatase domains (PTPc). A consensus carbonic anhydrase alpha-related domain (CAH) and a fibronectin type III domain (FN) are shared by these three variants. Positions of nine potential *N*-linked glycosylation sites and those of several *O*-GalNAc modifications^51^ are not indicated. (**b**) The samples of 1% Triton-soluble fractions prepared from adult WT cortex were immunoprecipitated with R-10G. R-10G-bound and R-10G-unbound materials were immunoblotted and probed with either Ptprz-S^34^ or R-10G antibody. (**c**) The RNA-seq transcriptome results of the gene of phosphacan/PTPRZ in indicated cell types are shown^49^. (**d**) The samples of 1% Triton-soluble fractions prepared from the cerebral cortex of adult WT mice and GlcNAc6ST1, 2, 3, and 4 KO mice were immunoprecipitated with R-10G. The R-10G-bound materials were immunoblotted and probed with the Ptprz-S antibody. Representative results are shown (n = 2 in **b**,**d**). Full-length blot images are presented in Supplementary Fig. S8.

Next, we asked which cerebral CSPG is modified with the R-10G-reactive KS in adulthood. Samples of 1% Triton-soluble fractions prepared from the cerebral cortex of adult mice were pre-treated with chondroitinase ABC and then immunoprecipitated with R-10G. The R-10G-immunoprecipitated proteins with a size of >300 kDa were molecularly identified with an in-gel trypsin digestion followed by LC-MS/MS (Figs 4a and S7). Phosphacan is a secreted CS-modified isoform of protein-tyrosine phosphatase receptor type Z (PTPRZ, also known as PTP-ζ or RPTPß). PTPRZ was identified in the immunoprecipitated samples of WT and GlcNAc6ST2-KO mice expressing the R-10G-positive KS. PTPRZ was not identified in the samples of GlcNAc6ST3-KO mice (Fig. S7, Table S1). Phosphacan carries both CS and KS with MW > 500 kDa^31–33^ and is distributed extracellularly in a diffuse manner^5^. The core protein of phosphacan has a molecular weight of 300 kDa^34^. Phosphacan/PTPRZ was a strong candidate for the R-10G reactive KS/CSPG expressed in the adult brain. Immunoblotting with the Ptprz-S antibody^34^ for the R-10G-immunoprecipitated proteins confirmed that phosphacan/PTPRZ is one of the KS/CSPGs (Fig. 4b). A majority of the Ptprz-S antibody-reactive molecules were present in the R-10G-unbound fraction and thus did not react with R-10G (Figs 4b and S8). The mouse phosphacan/PTPRZ gene, *Ptprz1*, is highly expressed in both astrocytes and OPCs of the adult brain cortex (Fig. 4c). It is conceivable that phosphacan and other PTPRZ isoforms expressed in astrocytes and neurons, and certain PTPRZ variants with low molecular weights^35^ in OPCs may account for the R-10G-negative forms present in the R-10G-unbound fractions of the adult mouse brain. Distribution of these R-10G-negative PTPRZ variants within the brain is unknown. We detected the reactivity of the Ptprz-S antibody on the R-10G-immunoprecipitated samples, which corresponded to a molecular weight of >300 kDa (Fig. 4d). It is an indication that this Ptprz-S reactivity is due to phosphacan and the ectopic domain of the full-length PTPRZ (PTPRZ-FL), but not due to the short form of PTPRZ^36^. The core protein of PTPRZ-FL (380 kDa) is hardly detected in the adult mouse brain since PTPRZ-FL is constitutively shed. The ectodomain is proteolytically released and has almost the same structure as phosphacan with a 300 kDa core protein^34^. Reactivity of the Ptprz-S antibody was negligible in the GlcNAc6ST3-KO sample (Fig. 4d). These results indicate that GlcNAc6ST3 is a major KS enzyme in the adult brain. It is likely that GlcNAc6ST3 in OPCs and oligodendrocytes post-translationally modifies a portion of phosphacan and the ectodomain of PTPRZ-FL that are then translocated into the vicinity of PV-positive and WFA-positive neurons. Phosphacan was earlier shown to bind NCAM and tenascin-R^37,38^. It is plausible that the R-10G-positive KS-modified phosphacan and the ectodomain of PTPRZ-FL are involved in maintaining the lattice-like PNNs by binding to NCAM or tenascin-R^39^ at the area of close proximity to the neuronal cell surface^40^. Possible involvement of the oligodendrocyte-derived GlcNAc6ST3, R-10G-positive KS phosphacan and the ectodomain of PTPRZ-FL in neuronal plasticity in the adult brain can be a subject of further study employing cell-type specific deletions of *Ptprz1* in GlcNAc6ST3-KO mice. It would be intriguing to analyze if modification of the R-10G-positive KS glycan could control the susceptibility of phosphacan and PTPRZ-FL to proteolytic cleavage mediated by plasmin and metalloproteinases^34,35^, and whether the R-10G-positive KS glycan influences the *cis* interaction of these PTPRZ core proteins on the cell surface by altering their affinity to galectin^41^ and pleiotrophin^42^. Another important question is whether the KS glycan could regulate the receptor functions of phosphacan and the ectodomain of PTPRZ-FL for contactin^43,44^, IL-34^45^ and pleiotrophin^42,46^.

The findings of the present study, together with our previous findings, indicates that GlcNAc6ST1 and GlcNAc6ST3 are the most important KS enzymes in the brain; the former temporally contributes to KS synthesis during the developmental stage, whereas the latter is the main KS enzyme in adulthood. Oligodendrocytes are a major source of the R-10G-positive KS in the adult brain. The possible contribution of oligodendrocyte subsets to the formation of PNNs and differentiation of PV-positive neurons through secretion of the R-10G-positive KS phosphacan and the ectopic domain of PTPRZ-FL will be investigated in the future.

## Materials and Methods

### Antibodies and enzymes

The following materials were obtained commercially from the indicated sources. Endo-ß-galactosidase (*E. freundii*), keratanase (*Pseudomonas sp*.), keratanase II (*Bacillus sp*.), and chondroitinase ABC (*P. vulgaris*) were from Seikagaku Corporation (Tokyo, Japan); The R-10G anti-GlcNAc-6-sulfated KS antibody^18,19^ was from Cosmo Bio (Tokyo, Japan); mouse anti-ß-actin antibody was from Sigma (St. Louis, MO); horseradish peroxidase (HRP)-conjugated goat anti-mouse IgG1 was from Caltag (Burlingame, CA); HRP-conjugated goat anti-rabbit IgG (H + L) was from Cell Signaling Technology (Danvers, MA); rabbit anti-GLAST and rabbit anti-synaptophysin (SYP) were from Frontier Institute Co., Ltd. (Hokkaido, Japan); rabbit anti-parvalbumin (PV) was from Abcam (Cambridge, UK); Alexa488-conjugated goat anti-rabbit IgG (H + L), Cy™3-conjugated goat anti-mouse IgG1, and HRP-conjugated goat anti-mouse IgG (H + L) were from Jackson ImmunoResearch Laboratories (West Grove, PA); Fluorescein isothiocyanate (FITC)-conjugated *Wisteria floribunda* lectin (WFA) was from Vector Laboratories, Inc. (Burlingame, CA); NeuroTrace™ Fluorescent Nissl Stain was from Thermo Fisher Scientific (Waltham, MA).

### Mice

C57BL/6 J mice were purchased from SLC Inc. (Hamamatsu, Japan). GlcNAc6ST1-deficient (KO)(*Chst2*^*−/−*^), and GlcNAc6ST2-KO (*Chst4*^*−/−*^) mice^22,47^, and GlcNAc6ST3-KO (*Chst5*^*−/−*^) mice^26^ were maintained on a C57BL/6 J genetic background. GlcNAc6ST4-KO (*Chst7*^*−/−*^) mice were generated by an *in vitro* fertilization at the Nagoya University Animal Care Department by obtaining the frozen sperm of the *Chst7*^*−/−*^ mice originated from the trans-NIH Knockout Mouse Project (KOMP). Genomic organization and targeting strategy of the mouse *Chst7* locus is shown in Fig. S3. Genotyping primers for GlcNAc6ST4-KO mice were used: Chst7-F1 forward primer: 5′- CACCCAACATTGAGGGAGAC -3′, Chst7-R1 reverse primer: 5′- CAGGTGGCATGCACATAGAT -3′, and Chst7KO-R1 reverse primer: 5′- ATCTGAGTTGCTGGCTTGGT -3′. Chst7-F1 and Chst7-R1 primer set amplified the sequence of the endogenous mouse *Chst7* allele and yielded a PCR product of 0.44 kb. Chst7-F1 and Chst7KO-R1 primer set amplified the sequence of the KO cassette-inserted allele and yielded a PCR product of 0.25 kb. Males and females of all genotypes at 2- to 6-month old were used for experiments. All mice were maintained under controlled SPF environmental conditions and provided with standard nourishment and water in the animal facilities of Nagoya University Graduate School of Medicine and Research Institute of Environmental Medicine. All experiments were approved by the Animal Research Committee of Nagoya University, and performed in accordance with the guidelines of Nagoya University.

### Mouse tissues

Mice were anesthetized and then transcardially perfused with phosphate buffered saline (PBS). Brains were dissected out and divided into sagittal parts. Regional parts of hemi-brains, namely, brainstem, thalamus, hippocampus, cerebellum, olfactory bulb, and cerebral cortex, were separated on ice, snap-frozen, and then stored at −80 °C for biochemical analysis. Hemi-brains were post-fixed overnight in phosphate buffer (PB) containing 4% paraformaldehyde, equilibrated into 30% sucrose in PBS and then embedded into Tissue-Tek® (O.C.T. compounds; Sakura, Torrance, CA) for frozen sectioning.

### Fractionation and enzymatic treatment of brain samples

Snap-frozen brain samples (~20 mg) were homogenized with a Dounce homogenizer in 600 µL (30 volumes of the tissue weight) of cold Tris-buffered saline (TBS) containing 1% Triton X-100 (w/v) and cOmplete™ protease inhibitor cocktail (Roche). The homogenized samples were placed on ice for 30 min. Supernatants were collected by centrifugation at 10,000 × *g* for 15 min at 4 °C. The supernatants were used as the 1% Triton-soluble fraction. The protein concentration was measured by the Bradford method. For pretreatment, 1% Triton-soluble fractions were heated for 10 min at 95 °C. Supernatants were collected by centrifugation at 10,000 × *g* for 3 min at 4 °C. The supernatants were treated with Endo-ß-galactosidase (10 µU/µl), keratanase (1 mU/µl), or keratanase II (50 µU/µl) at 37 °C overnight. In the case of treatment with chondroitinase ABC (1 mU/µl), the reaction time was 3 h. The enzymatic reaction was stopped by heating the samples at 95 °C for 5 min.

### Immunoblot

The proteins (20 µg) were separated using 7.5% polyacrylamide gel electrophoresis (SuperSep, WAKO, Osaka, Japan) and blotted onto a polyvinylidene difluoride (PVDF) membrane (GE Healthcare Life Sciences). The membrane was blocked with 5% skim milk in TBS containing 0.1% Tween-20 (TBS-T) for 1 h at room temperature and then incubated at 4 °C overnight with primary antibody: R-10G anti-KS antibody (1:200 dilution) or the rabbit polyclonal anti-Ptprz-S antibody (1:500 dilution)^34^ in 5% skim milk/TBS-T overnight at 4 °C. Membranes were washed with TBS-T and incubated for 30 min at room temperature with HRP-conjugated secondary antibodies. Bound antibodies were detected with a Novex ECL Chemiluminescent reagent kit (Thermo Fisher Scientific) and an Amersham Imager 600 (GE Healthcare Life Sciences). Densitometry analysis of immunoreactive bands was performed with the ImageJ software (http://imagej.nih.gov/ij/) (National Institutes of Health, Bethesda, MD).

### Immunoprecipitation

The proteins (200 µg) in the 1% Triton-soluble fraction of the cerebral cortex were mixed with a complex of the R-10G anti-KS antibody and Protein G Dynabeads™ (Thermo Fisher Scientific) in PB containing 0.02% Tween-20 (PB-T) for 30 min at room temperature. The immunocomplexes bound to the Protein G Dynabeads™ were isolated with the DynaMag™ -2 Magnet (Thermo Fisher Scientific). Pretreatment of the samples with chondroitinase ABC was performed in the case of immunoprecipitation followed by the R-10G immunoblot, and molecular identification with liquid chromatography-tandem mass spectrometry (LC-MS/MS).

### Identification of KS-modified proteins by LC-MS/MS analyses

For LC-MS/MS analyses, polyacrylamide gel electrophoresis separation and an in-gel trypsin digestion of the R-10G immunoprecipitated glycoproteins with MW > 300 kDa were performed as described previously^48^. The resultant digested peptides were reconstituted in 0.1% formic acid and analyzed by a Thermo Orbitrap Elite mass spectrometer (Thermo Fisher Scientific). The peptides were directly infused into the ESI source through a packed nano-capillary column (NTCC-360/75-3; Nikkyo Technos Co. Ltd., Tokyo, Japan) equilibrated in 0.1% formic acid at a flow rate of 300 nL/min, and were sequentially eluted with an acetonitrile gradient of 0–30% for 10 min, 30–80% for 2 min, followed by an 8-min hold at 80%. The spectrometer was operated in a data-dependent mode using the normalized collision energy of 30. The temperature of the ion transfer tube was set at 250 °C. The spray voltage was at 2.0 kV. The full mass spectra were acquired using an *m/z* range of 350–2000. The resultant MS and MS/MS data were searched against the NCBI database using the Mascot ver.2.5.1 (Matrixscience, UK) with Proteome Discoverer software (Thermo Fisher Scientific).

### Immunohistochemistry

Frozen brain tissues were cut into 10-µm-thick sections on a cryostat and collected on MAS-coated glass slides (SF17293; Matsunami, Osaka, Japan). Sections were air-dried for 30 min, rinsed with PBS to remove O.C.T. compounds, and then blocked in PBS containing 5% normal goat serum and 0.3% Triton-X 100 for 1 h at room temperature. Sections were incubated with R-10G (1:100 dilution), and rabbit anti-GLAST (1:1,000), rabbit anti-SYP (1:200), rabbit anti-PV (1:200), or FITC-conjugated WFA (1:100) in PBS containing 0.03% Triton-X 100 at 4 °C overnight. Sections were washed with PBS and then incubated with Cy3-anti-mouse IgG1 (1:400 dilution) and Alexa488-conjugated goat anti-rabbit IgG (H + L) (1:500) for 30 min at room temperature. After washing with PBS, sections were incubated with Hoechst 33342 solution (Dojindo, Japan; 1:1,000 dilution) for 5 min at room temperature for staining cell nuclei, or NeuroTrace™ 435/455 blue fluorescent Nissl stain for visualizing neurons. Stained sections were mounted in FluorSave™ Reagent (Merck, Darmstadt, Germany). Signals were visualized and captured by a fluorescent microscope (model BX50, Olympus) at the same exposure setting for each antibody, or a confocal microscope.

### Confocal microscopy

Cryostat-cut brain sections (10-μm-thick) from adult mouse brains were co-stained as described above. The signals were visualized with a total internal reflection fluorescence/confocal laser scanning microscope (A1Rsi, Nikon, Tokyo, Japan). The Z-step scans were recorded (0.2 μm/step, steps = 51). The images were 3D-reconstructed and analyzed by NIS-Elements analysis software (Nikon).

### Gene expression patterns in adult brain cells

Data pertinent to the genes of GlcNAc6STs and phosphacan were mined from a published RNA sequencing (RNA-Seq) analysis of purified neurons, oligodendrocyte precursor cells, newly formed oligodendrocytes, myelinating oligodendrocytes, astrocytes, microglia, and endothelial cells from adult mouse brains^49^. Comparison of their transcription profiles in various cell types of the brain was performed with an RNA-Seq transcriptome platform (http://web.stanford.edu/group/barres_lab/brain_rnaseq.html). A value of fragments per kilobase of transcript sequence per million mapped fragments (FPKM) of ~0.04 was determined as a threshold for minimum gene expression^49^.

### Statistical analysis

Values were analyzed via the one-way analysis of variance with Tukey’s test (Fig. 2) by using Prism software (GraphPad Software, La Jolla, CA, USA). Differences were regarded as significant when *P* < 0.05. All data are means ± SD unless otherwise noted.

## Supplementary information

Supplementary Information

Table S1

## References

[1] Dityatev A, Schachner M (2003). Extracellular matrix molecules and synaptic plasticity. Nat Rev Neurosci.

[2] Miyata S, Kitagawa H (2015). Mechanisms for modulation of neural plasticity and axon regeneration by chondroitin sulphate. J Biochem.

[3] Yamaguchi Y (2000). Lecticans: organizers of the brain extracellular matrix. Cell Mol Life Sci.

[4] Djerbal L, Lortat-Jacob H, Kwok J (2017). Chondroitin sulfates and their binding molecules in the central nervous system. Glycoconj J.

[5] Deepa SS (2006). Composition of perineuronal net extracellular matrix in rat brain: a different disaccharide composition for the net-associated proteoglycans. J Biol Chem.

[6] Carulli D (2010). Animals lacking link protein have attenuated perineuronal nets and persistent plasticity. Brain.

[7] Hockfield S, Kalb RG, Zaremba S, Fryer H (1990). Expression of neural proteoglycans correlates with the acquisition of mature neuronal properties in the mammalian brain. Cold Spring Harb Symp Quant Biol.

[8] Celio MR, Blumcke I (1994). Perineuronal nets–a specialized form of extracellular matrix in the adult nervous system. Brain Res Brain Res Rev.

[9] Lander C, Kind P, Maleski M, Hockfield S (1997). A family of activity-dependent neuronal cell-surface chondroitin sulfate proteoglycans in cat visual cortex. J Neurosci.

[10] Celio MR, Spreafico R, De Biasi S, Vitellaro-Zuccarello L (1998). Perineuronal nets: past and present. Trends Neurosci.

[11] Sorg BA (2016). Casting a Wide Net: Role of Perineuronal Nets in Neural Plasticity. J Neurosci.

[12] Takeda-Uchimura Y (2015). Requirement of keratan sulfate proteoglycan phosphacan with a specific sulfation pattern for critical period plasticity in the visual cortex. Exp Neurol.

[13] Funderburgh JL (2002). Keratan sulfate biosynthesis. IUBMB Life.

[14] Uchimura K (2015). Keratan sulfate: biosynthesis, structures, and biological functions. Methods Mol Biol.

[15] Finne J, Krusius T, Margolis RK, Margolis RU (1979). Novel mannitol-containing oligosaccharides obtained by mild alkaline borohydride treatment of a chondroitin sulfate proteoglycan from brain. J Biol Chem.

[16] Krusius T, Finne J, Margolis RK, Margolis RU (1986). Identification of an O-glycosidic mannose-linked sialylated tetrasaccharide and keratan sulfate oligosaccharides in the chondroitin sulfate proteoglycan of brain. J Biol Chem.

[17] Krusius T, Reinhold VN, Margolis RK, Margolis RU (1987). Structural studies on sialylated and sulphated O-glycosidic mannose-linked oligosaccharides in the chondroitin sulphate proteoglycan of brain. Biochem J.

[18] Kawabe K (2013). A novel antibody for human induced pluripotent stem cells and embryonic stem cells recognizes a type of keratan sulfate lacking oversulfated structures. Glycobiology.

[19] Nakao H (2017). Binding specificity of R-10G and TRA-1-60/81, and substrate specificity of keratanase II studied with chemically synthesized oligosaccharides. Glycoconj J.

[20] Uchimura K, Rosen SD (2006). Sulfated L-selectin ligands as a therapeutic target in chronic inflammation. Trends Immunol.

[21] Habuchi H (2000). The occurrence of three isoforms of heparan sulfate 6-O-sulfotransferase having different specificities for hexuronic acid adjacent to the targeted N-sulfoglucosamine. J Biol Chem.

[22] Uchimura K (2005). A major class of L-selectin ligands is eliminated in mice deficient in two sulfotransferases expressed in high endothelial venules. Nat Immunol.

[23] Hoshino H (2014). KSGal6ST is essential for the 6-sulfation of galactose within keratan sulfate in early postnatal brain. J Histochem Cytochem.

[24] Hemmerich S (2001). Sulfation of L-selectin ligands by an HEV-restricted sulfotransferase regulates lymphocyte homing to lymph nodes. Immunity.

[25] Lee JK, Bhakta S, Rosen SD, Hemmerich S (1999). Cloning and characterization of a mammalian N-acetylglucosamine-6-sulfotransferase that is highly restricted to intestinal tissue. Biochem Biophys Res Commun.

[26] Hayashida Y (2006). Matrix morphogenesis in cornea is mediated by the modification of keratan sulfate by GlcNAc 6-O-sulfotransferase. Proc Natl Acad Sci USA.

[27] Foyez T (2015). Microglial keratan sulfate epitope elicits in central nervous tissues of transgenic model mice and patients with amyotrophic lateral sclerosis. Am J Pathol.

[28] Yagi H (2005). Development of structural analysis of sulfated N-glycans by multidimensional high performance liquid chromatography mapping methods. Glycobiology.

[29] Uchimura K (2002). Specificities of N-acetylglucosamine-6-O-sulfotransferases in relation to L-selectin ligand synthesis and tumor-associated enzyme expression. J Biol Chem.

[30] Zhang Z (2017). Deficiency of a sulfotransferase for sialic acid-modified glycans mitigates Alzheimer’s pathology. Proc Natl Acad Sci USA.

[31] Faissner A (1994). Isolation of a neural chondroitin sulfate proteoglycan with neurite outgrowth promoting properties. J Cell Biol.

[32] Garwood J (1999). DSD-1-proteoglycan is the mouse homolog of phosphacan and displays opposing effects on neurite outgrowth dependent on neuronal lineage. J Neurosci.

[33] Rauch U (1991). Isolation and characterization of developmentally regulated chondroitin sulfate and chondroitin/keratan sulfate proteoglycans of brain identified with monoclonal antibodies. J Biol Chem.

[34] Chow JP, Fujikawa A, Shimizu H, Suzuki R, Noda M (2008). Metalloproteinase- and gamma-secretase-mediated cleavage of protein-tyrosine phosphatase receptor type Z. J Biol Chem.

[35] Chow JP, Fujikawa A, Shimizu H, Noda M (2008). Plasmin-mediated processing of protein tyrosine phosphatase receptor type Z in the mouse brain. Neurosci Lett.

[36] Nishiwaki T, Maeda N, Noda M (1998). Characterization and developmental regulation of proteoglycan-type protein tyrosine phosphatase zeta/RPTPbeta isoforms. J Biochem.

[37] Milev P (1994). Interactions of the chondroitin sulfate proteoglycan phosphacan, the extracellular domain of a receptor-type protein tyrosine phosphatase, with neurons, glia, and neural cell adhesion molecules. J Cell Biol.

[38] Milev P (1998). High affinity binding and overlapping localization of neurocan and phosphacan/protein-tyrosine phosphatase-zeta/beta with tenascin-R, amphoterin, and the heparin-binding growth-associated molecule. J Biol Chem.

[39] Celio MR, Chiquet-Ehrismann R (1993). ‘Perineuronal nets’ around cortical interneurons expressing parvalbumin are rich in tenascin. Neurosci Lett.

[40] Galtrey CM, Fawcett JW (2007). The role of chondroitin sulfate proteoglycans in regeneration and plasticity in the central nervous system. Brain Res Rev.

[41] Abbott KL, Matthews RT, Pierce M (2008). Receptor tyrosine phosphatase beta (RPTPbeta) activity and signaling are attenuated by glycosylation and subsequent cell surface galectin-1 binding. J Biol Chem.

[42] Kuboyama K, Fujikawa A, Suzuki R, Tanga N, Noda M (2016). Role of Chondroitin Sulfate (CS) Modification in the Regulation of Protein-tyrosine Phosphatase Receptor Type Z (PTPRZ) Activity: Pleiotrophin-Ptprz-A Signaling is Involved in Oligodendrocyte Differentiation. J Biol Chem.

[43] Peles E (1995). The carbonic anhydrase domain of receptor tyrosine phosphatase beta is a functional ligand for the axonal cell recognition molecule contactin. Cell.

[44] Lamprianou S, Chatzopoulou E, Thomas JL, Bouyain S, Harroch S (2011). A complex between contactin-1 and the protein tyrosine phosphatase PTPRZ controls the development of oligodendrocyte precursor cells. Proc Natl Acad Sci USA.

[45] Nandi S (2013). Receptor-type protein-tyrosine phosphatase zeta is a functional receptor for interleukin-34. J Biol Chem.

[46] Maeda N, Nishiwaki T, Shintani T, Hamanaka H, Noda M (1996). 6B4 proteoglycan/phosphacan, an extracellular variant of receptor-like protein-tyrosine phosphatase zeta/RPTPbeta, binds pleiotrophin/heparin-binding growth-associated molecule (HB-GAM). J Biol Chem.

[47] Uchimura K (2004). N-acetylglucosamine 6-O-sulfotransferase-1 regulates expression of L-selectin ligands and lymphocyte homing. J Biol Chem.

[48] Yagi H (2008). Neural complex-specific expression of xylosyl N-glycan in Ciona intestinalis. Glycobiology.

[49] Zhang Y (2014). An RNA-sequencing transcriptome and splicing database of glia, neurons, and vascular cells of the cerebral cortex. J Neurosci.

[50] Bartels MF (2016). Protein O-Mannosylation in the Murine Brain: Occurrence of Mono-O-Mannosyl Glycans and Identification of New Substrates. PLoS One.

[51] Trinidad JC, Schoepfer R, Burlingame AL, Medzihradszky KF (2013). N- and O-glycosylation in the murine synaptosome. Mol Cell Proteomics.

