# Supplementary material for: GlcNAc6ST3 is a keratan sulfate sulfotransferase for the protein-tyrosine phosphatase PTPRZ in the adult brain
Source: Sci Rep. 2019 Mar 13;9:4387. doi: 10.1038/s41598-019-40901-2 (PMC6416290; doi:10.1038/s41598-019-40901-2)
Supplement: Supplementary file 1 — Supplementary Information [file 41598_2019_40901_MOESM1_ESM.docx]

**Narentuya et al.**

**SUPPLEMENTARY INFORMATION for**

**GlcNAc6ST3 is a keratan sulfate sulfotransferase for the protein-tyrosine phosphatase PTPRZ in the adult brain**

Narentuya, Yoshiko Takeda-Uchimura, Tahmina Foyez, Zui Zhang, Tomoya O. Akama, Hirokazu Yagi, Koichi Kato, Yukio Komatsu, Kenji Kadomatsu, and Kenji Uchimura*****

***Correspondence and requests for materials should be addressed to:**

Kenji Uchimura, Ph.D., Unité de Glycobiologie Structurale et Fonctionnelle, UMR 8576 CNRS, Université de Lille, 59655 Villeneuve d'Ascq, France. Email: kenji.uchimura@univ-lille.fr

**Figures S1-S8**

**Table S1**

**Supplementary Materials and Methods**

**
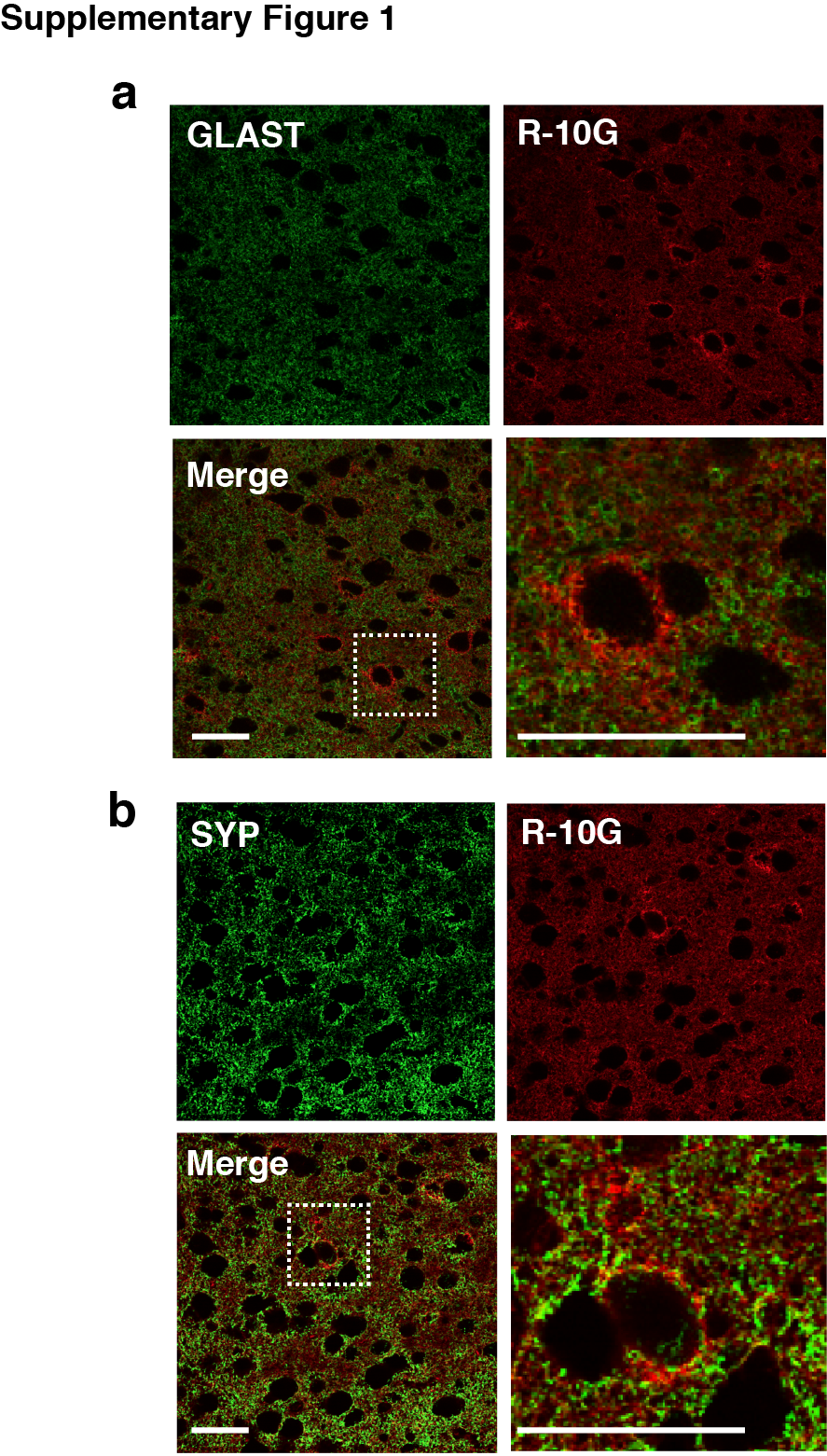
**

**Figure S1. Localization of the R-10G-reactive GlcNAc-6-sulfate KS in the cerebral cortex of adult mice.**

Brain sections from adult WT mice were immunostained with R-10G (*red*) and an anti-GLAST (*green*) (**a**) or an anti-synaptophysin (SYP) (*green*) (**b**). Representative confocal microscope images of the cerebral cortex are shown (n = 3). Magnifications of boxed regions in the merged panels are indicated. Scale bars: 20 µm.


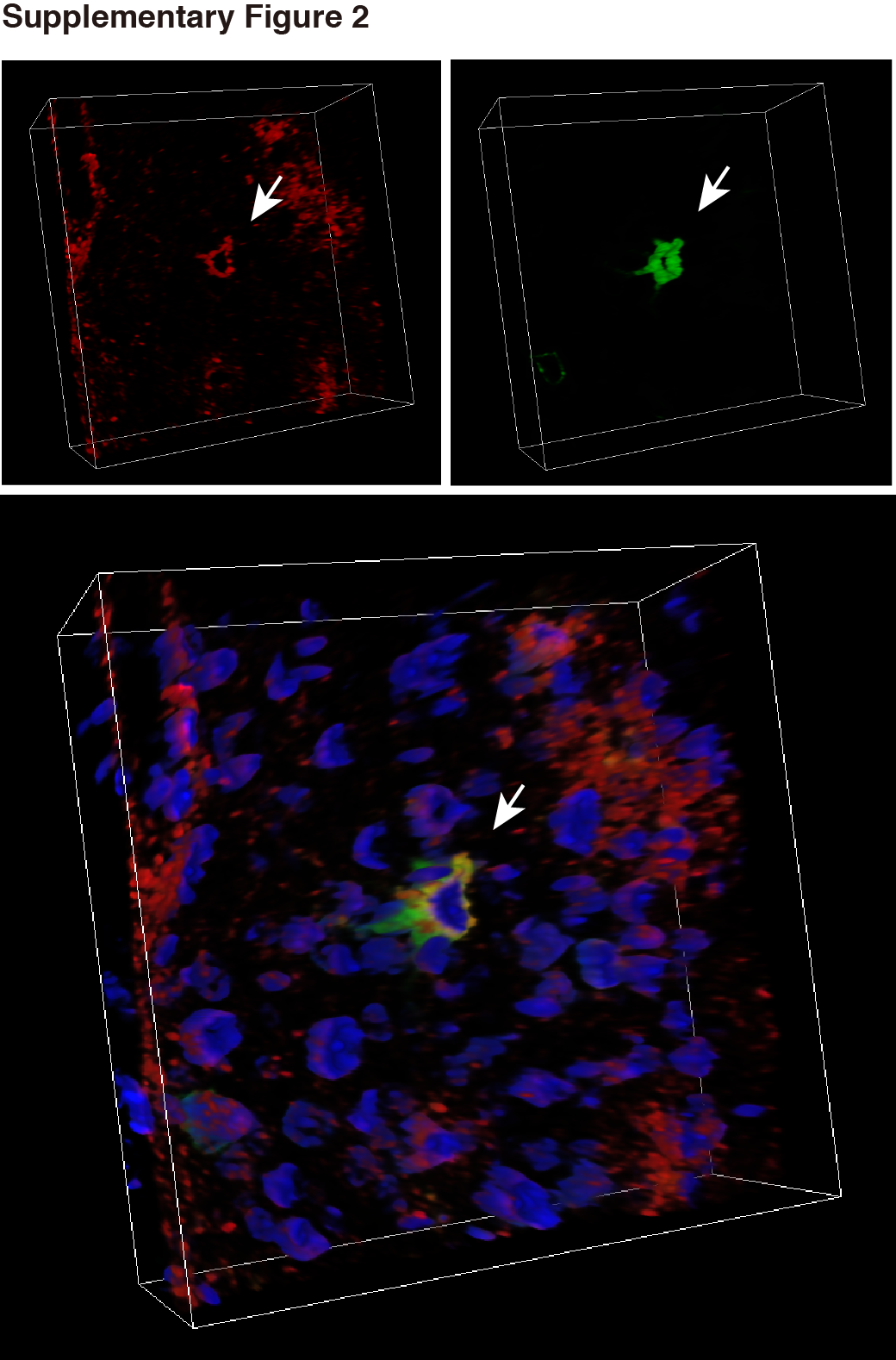


**Figure S2. Pericellular R-10G signals in close proximity to the soma of a WFA–positive neuron.**

A representative image of the Z-stack confocal microscopy of the area indicated in **Fig. 2c** is shown (width, 109.81 μm; height, 109.81 μm; depth, 10 μm). *Arrow* denotes dense pericellular R-10G signals (*red*) in close proximity to the NeuroTrace Nissl-stained soma (*blue*) of a WFA (*green*)-positive neuron.

**
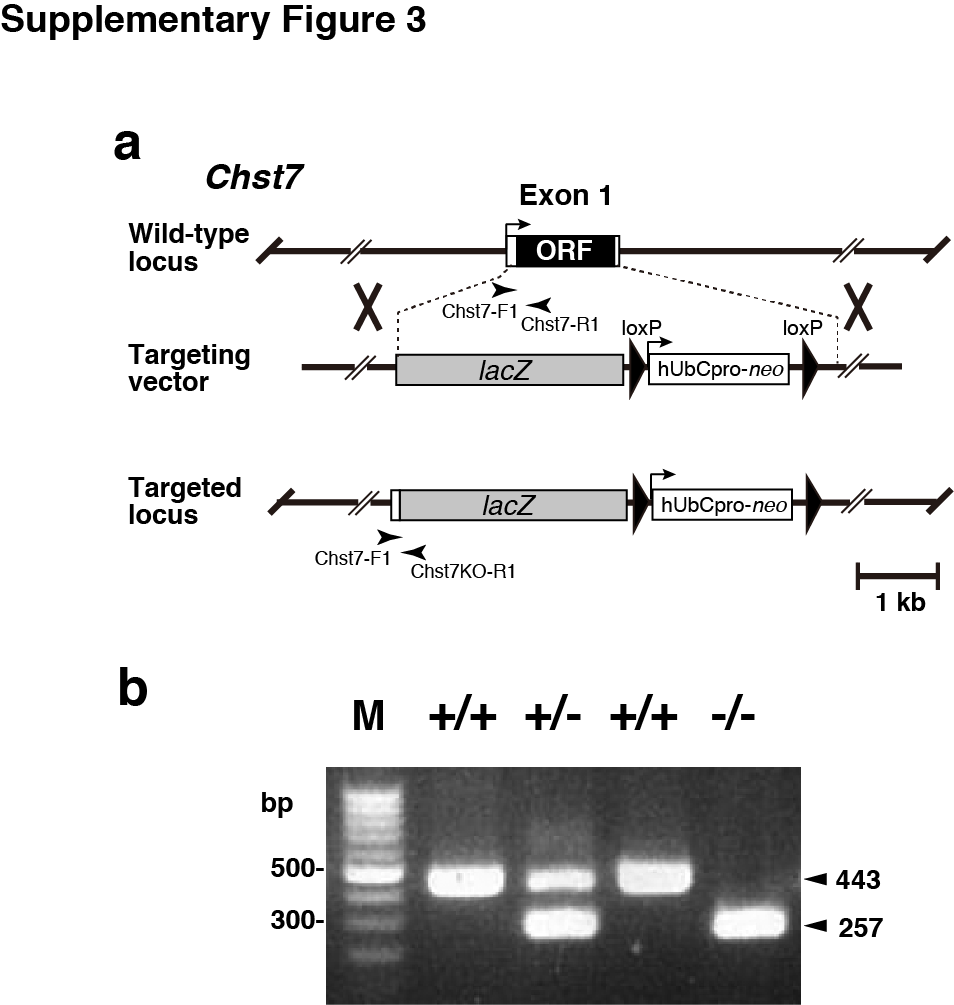
**

**Figure S3. Generation of GlcNAc6ST4-deficient mice.**

(**a**) Schematic diagram of the *Chst7* locus and the homologous recombination construct from the KOMP project. The targeting vector replaces the open reading frame (ORF) of the entire protein-coding region in the *Chst7* exon 1 with the cassette of the *E. coli* lacZ gene (*lacZ*) and the human ubiquitin C gene promoter (hUbCpro)/neomycin-resistant gene (*neo*), which is flanked by loxP. *Arrowheads* denote locations of three types of PCR primers used for genotyping. (**b**) Genotyping results for *Chst7* wild-type (+/+), heterozygous (+/-), and homozygous (-/-) mice.


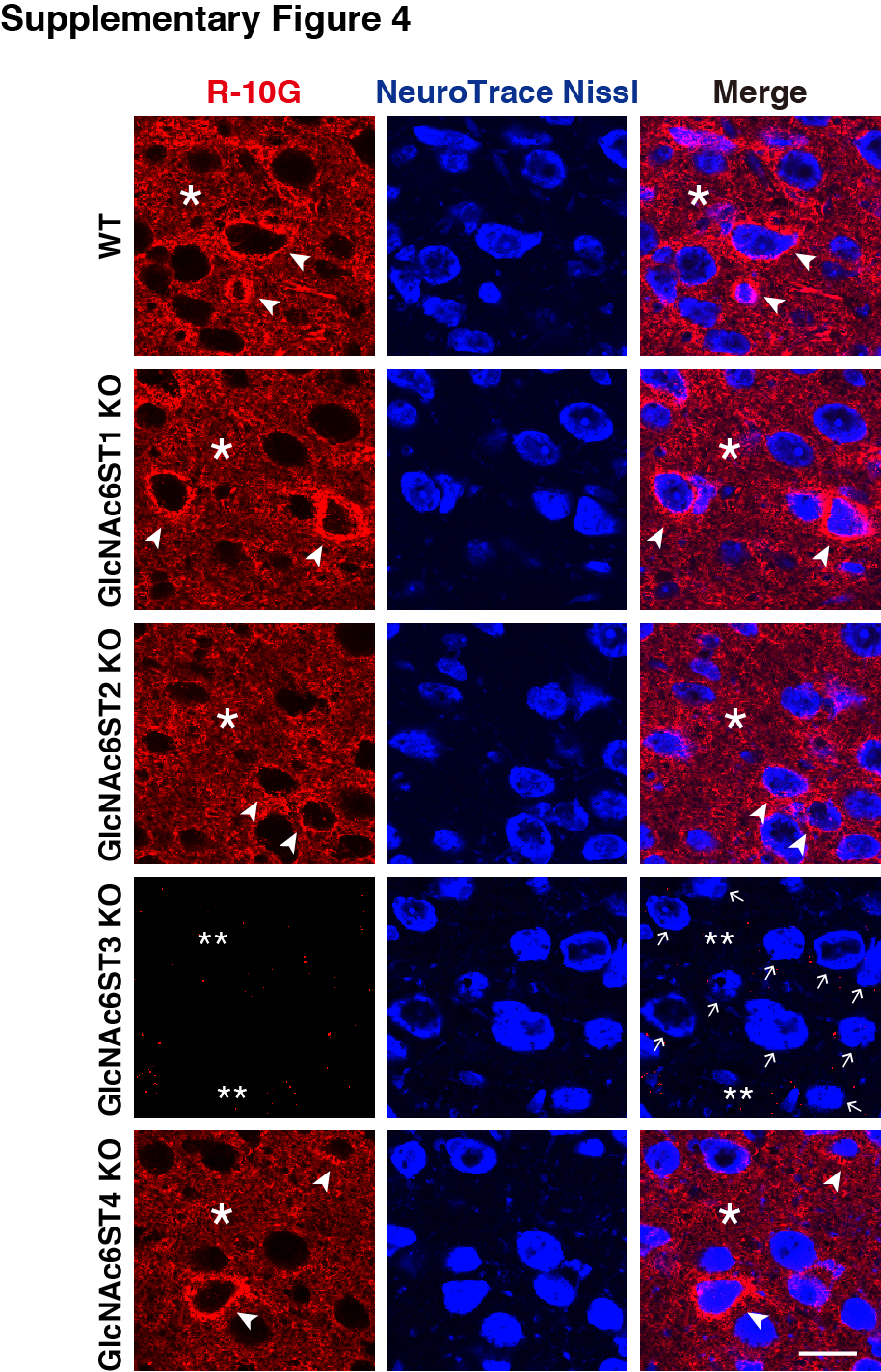


**Figure S4. R-10G staining signals in the pericellular regions and neuropils are negligible in the adult GlcNAc6ST3 KO brain.**

Brain sections from adult WT mice and GlcNAc6ST1, 2, 3, and 4 single KO mice were immunostained with R-10G (*red*) followed by NeuroTrace Nissl staining (*blue*). Representative confocal microscope images of the visual cortex are shown (n = 5 for WT; n = 4 for GlcNAc6ST1 KO; n = 3 for GlcNAc6ST2 KO; n = 5 for GlcNAc6ST3 KO; n = 3 for GlcNAc6ST4 KO). Dense R-10G staining signals in pericellular spaces (*arrowheads*) were deficient in GlcNAc6ST3 KO mice (*arrows*). Diffuse R-10G staining signals in intercellular spaces (*asterisks*) were deficient in GlcNAc6ST3 KO mice (*double asterisks*). Digital images were captured at the same setting for each staining. Scale bar: 10 µm.


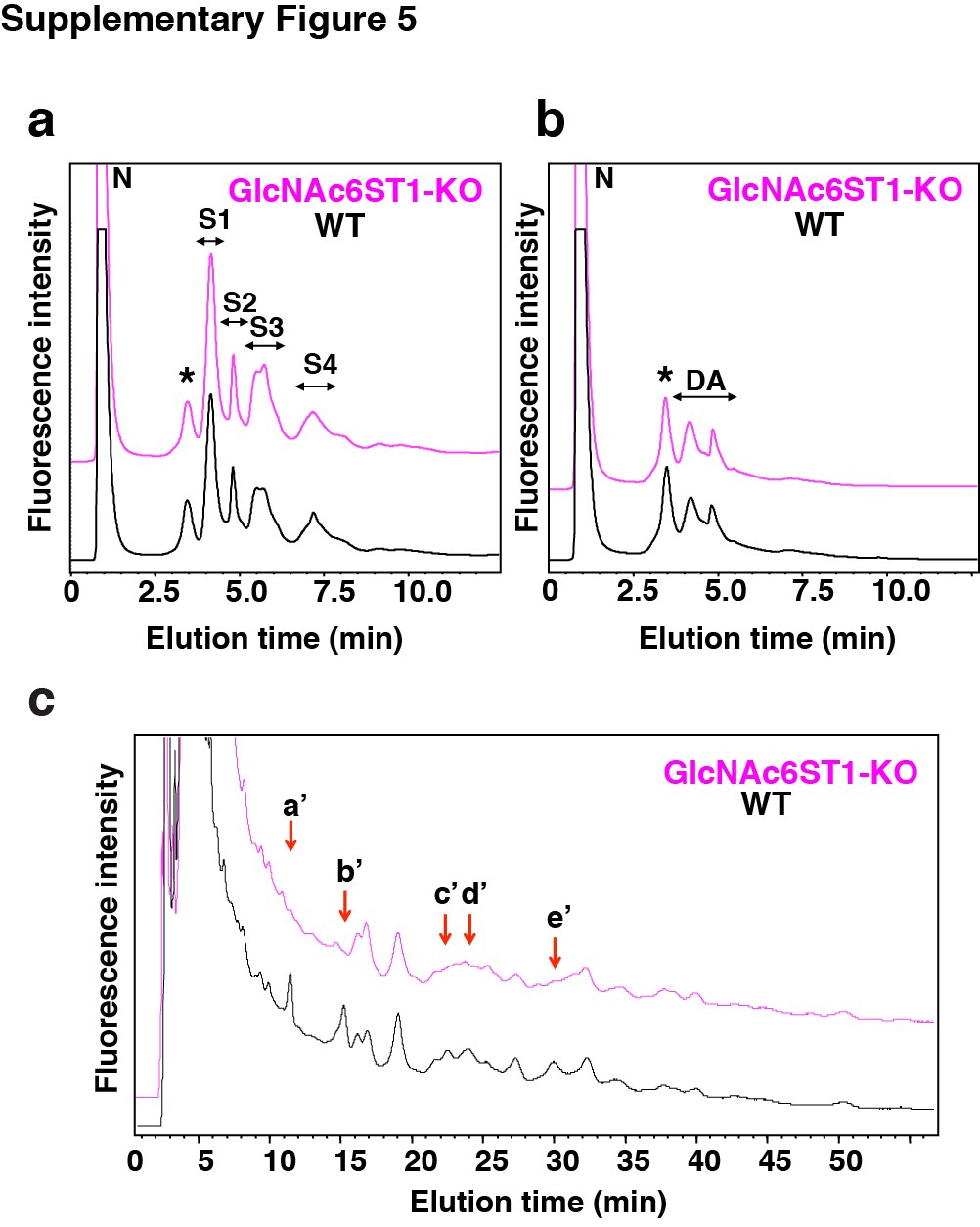


**Figure S5. Deficiency of GlcNAc-6-sulfated *N*-glycans in the GlcNAc6ST1-KO adult brain revealed by HPLC mapping/MALDI-TOF MS.**

(**a,b**) High performance liquid chromatography (HPLC) elution profiles of pyridyl-2-aminated (PA)-*N*-glycans derived from the adult brain of WT and GlcNAc6ST1-KO mice on a DEAE ion-exchange column before (**a**) and after (**b**) a sialidase treatment. S1, S2, S3, and S4 indicate the fractions that contain mono-, di-, tri-, and tetra-sialyl oligosaccharides, respectively. N and DA indicate the neutral and asialo-anionic fractions, respectively. *Asterisks* denote the fractions containing no detectable PA-oligosaccharides. (**c**) HPLC *N*-glycan elution profiles on an octadecyl silica (ODS) column of the asialo-anionic fractions separated by the DEAE column (“DA” in **a**). The *red arrows* indicate the positions of the peaks (a’-e’) that were not detected in the profile of GlcNAc6ST1-KO mouse brain.


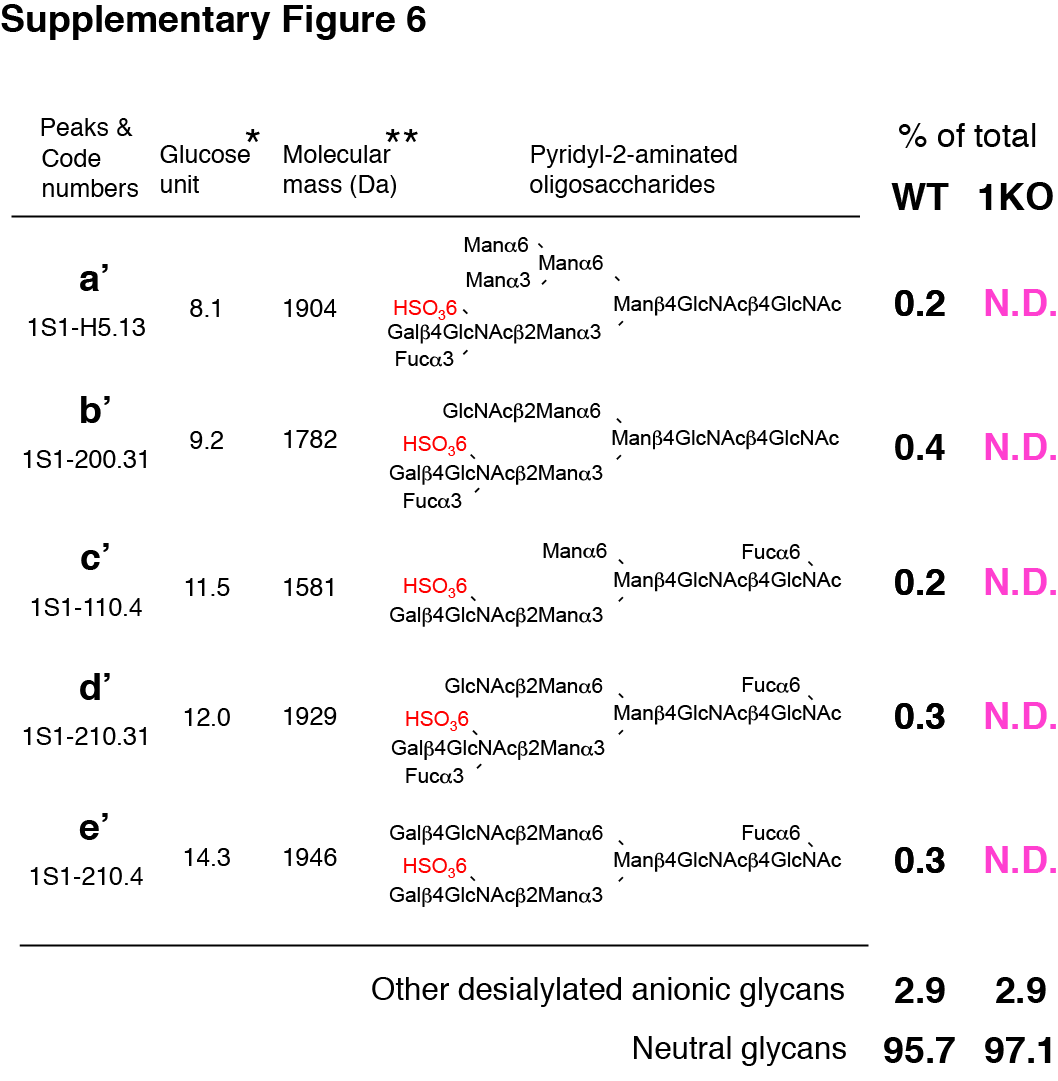


**Figure S6. GlcNAc6ST1-KO adult mouse brains are deficient in GlcNAc-6-sulfated *N*-glycan structures.**

Structures of pyridyl-2-aminated (PA)-*N*-glycans that were eluted using an ODS HPLC (peaks a’-e’ in **Fig S5c**) were identified. Molar ratio of the *N*-glycan content in the brain tissues of WT and GlcNAc6ST1 KO adult mice was calculated on the basis of peak areas detected by ODS HPLC for the N and DA fractions (**Fig S5**). GlcNAc6ST1 deficiency resulted in loss of GlcNAc(6S)ß1-2Manα1-3Man branch-containing *N*-glycans in the adult mouse brain (the five peaks corresponding to a’-e’ in **Fig S5c**). *glucose unit value on the ODS column. **Average mass calculated from the m/z values of [M-H]^-^ ions for PA-oligosaccharides.


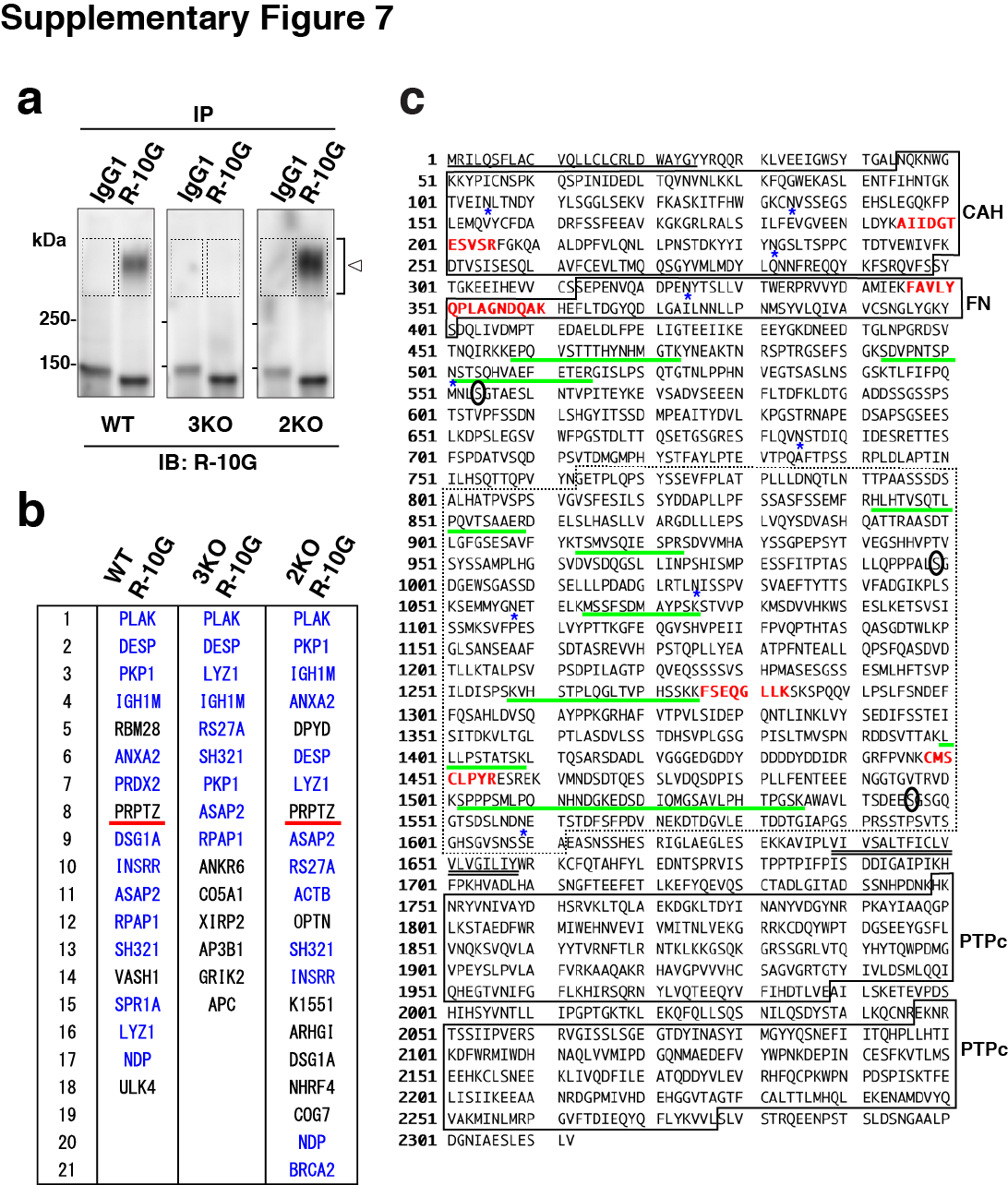


**Figure S7. Phosphacan/PTPRZ is identified in R-10G-immunoprecipitated materials prepared from the cerebral cortex of adult mice.**

(**a**) The R-10G immunoprecipitated proteins (MW > 300 kDa) prepared from the cerebral cortex of adult WT, GlcNAc6ST3-KO and GlcNAc6ST2-KO mice were subjected to the LC-MS/MS analysis. Mouse IgG1-immunoprecipitated proteins were used as negative controls. Western blotting with R-10G was confirmed that the samples prepared from the brains of WT and GlcNAc6ST2-KO mice, but not GlcNAc6ST3-KO mice, contained R-10G-reactive materials (*open arrowhead).* Proteins with MW > 300 kDa in each sample lane (*dashed boxes*) were isolated from the SDS-PAGE gel and subjected to an in-gel trypsin digestion as described in the Materials and Methods. (**b**) Protein names identified in the > 300-kDa R-10G immunoprecipitated samples of adult WT, GlcNAc6ST3-KO, and GlcNAc6ST2-KO mice. Proteins that were also identified in IgG1 controls of either genotype are indicated in *blue*. Only phosphacan/PTPRZ (PRPTZ) was identified in both WT and GlcNAc6ST2-KO mice, but not in GlcNAc6ST3-KO mice, which lack the most of the R-10G reactivity in the brains, or IgG1 controls (*red underline*). Keratins were excluded from the results. MASCOT search results were shown in **Table S1.** (**c**) LC-MS/MS peptide hits for phosphacan/PTPRZ are shown in the amino acid sequence of PTPRZ. Sequences of the observed peptides are indicated in *red*. The peptides with *O*-mannose modifications are underlined in *green* (Bartels et al., 2016, Trinidad et al., 2013). Positions of the CS GAG modification sites are marked (Nandi et al., 2013). *Asterisks* denote nine potential *N*-linked glycosylation sites. Consensus carbonic anhydrase alpha-related domain (CAH), a fibronectin type III domain (FN) and the dual phosphatase domains (PTPc) are *boxed*. The peptide stretch lacking in the short spliced isoform of PTPRZ is indicated as a *dashed box*. The transmembrane domain is marked as *double underline*. Full-length blot images are presented in Supplementary **Fig S8**.


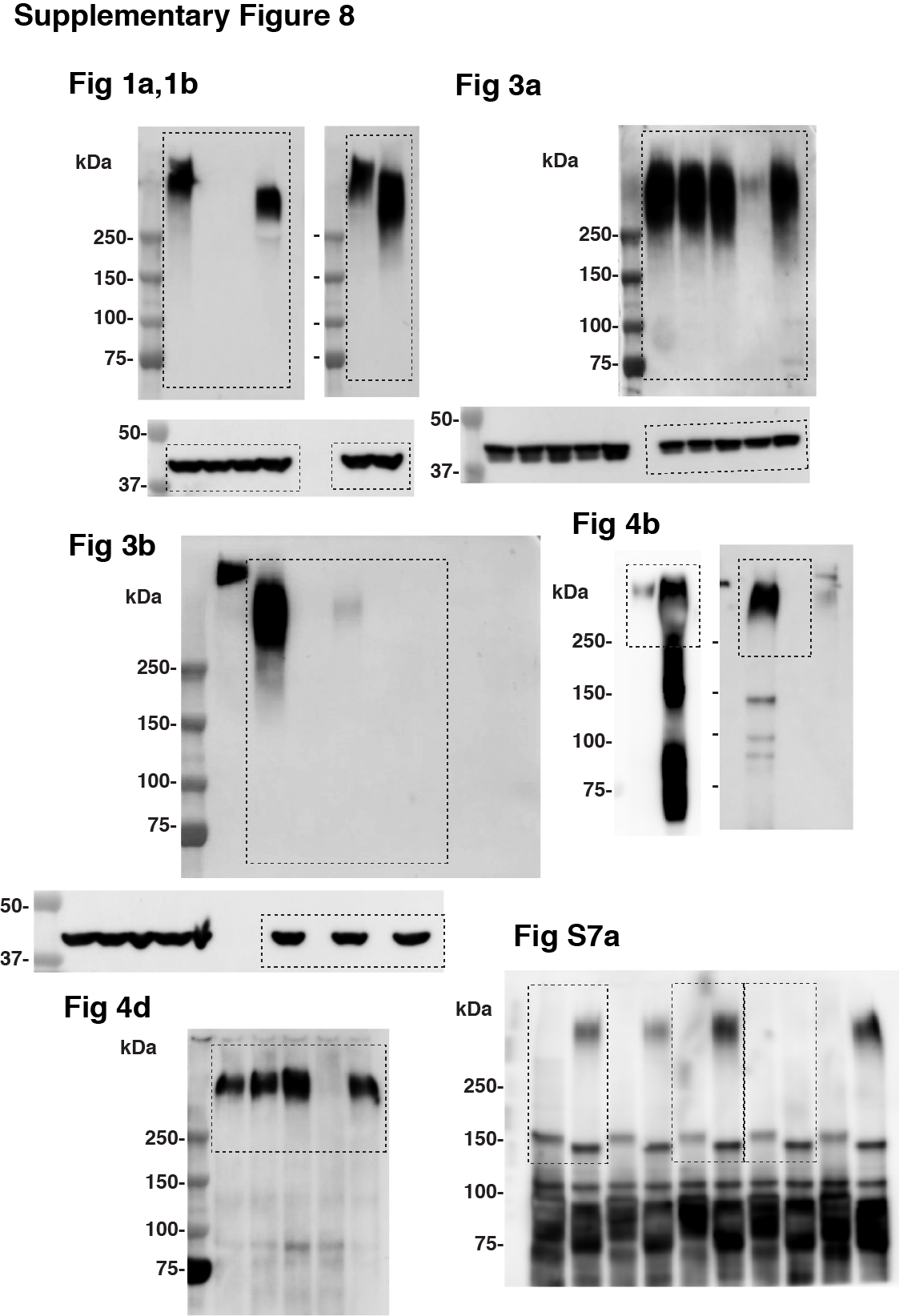


**Figure S8. Full-length blot images of those in Figs. 1, 3, 4 and S7.**

**SUPPLEMENTARY MATERIALS AND METHODS**

*Materials*

Materials used for the experiments were purchased from the sources indicated below. The α-mannosidase, β-galactosidase, and β-*N*-acetylhexosaminidase from jack bean were purchased from Seikagaku (Tokyo, Japan). The α-sialidase from *Arthrobacter ureafaciens* was from Nacalai Tesque (Kyoto, Japan). The pyridyl-2-aminated (PA) -derivatives of isomalto-oligosaccharides (with various degrees of polymerization) and the PA derivatives of *N*-linked oligosaccharides with the HPLC database code numbers H5.12, 1S1-100.41, 1S1-110.41, 1S1-210.31, and 1S1-210.4 were prepared in a previous study (Yagi et al., 2005).

*HPLC mapping*

All experimental procedures including delipidation of the adult brain samples, chromatographic conditions, glycosidase treatments, desulfation reactions, and matrix assisted laser desorption/ionization-time of flight-mass sepectrometry (MALDI-TOF MS) have been described previously (Nakagawa et al., 1995, Takahashi et al., 1995, Yagi et al., 2005) with the exception of slight modifications of purification of PA-glycans and separation of the glycans by an anion exchange column. Delipidated brain lysates were dried by lyophilization. The *N*-glycans in the dried samples were digested by hydrazinolysis under the conditions previously described (Yagi et al., 2005). Oligosaccharides were released from the hemi-brain extracts by heating with 0.2 ml of anhydrous hydrazine at 100 °C for 10 h in an evacuated sealed tube. On-column removal of the excess hydrazine, peptide materials, and detergents, and on-column *N*-acetylation were performed by using a carbon column (GL-Pak Carbograph, GL Sciences, Tokyo, Japan)(Tanabe & Ikenaka, 2006). The reducing ends of the *N*-glycans were labeled with 2-aminopyridine (PA). The PA-glycan mixture was then purified on a cellulose column. The mixture of PA-*N*-glycan derivatives was separated by HPLC on a mono-Q column (GE Healthcare Science) at 30 ^o^C at a flow rate of 1.0 ml/min using solvent gradient before and after sialidase treatments. The solvent A was aqueous ammonia (pH 9.0), and the solvent B was 50 mM ammonium acetate solution (pH 9.0). The column was equilibrated with the solvent A. After injection of the sample, the concentration of the solvent B was increased to 20% with a linear gradient in 30 min. These desialylated PA-*N*-glycans were individually separated and identified sequentially on a Shim-pack HRC-ODS column (Shimadzu, Kyoto, Japan). The identification of *N*-glycan structures was based on their elution positions on these columns in comparison to the PA-glycans in the database of GALAXY and to the existing HPLC data for sulfated oligosaccharides (Yagi et al., 2005). The structures of PA-glycans not registered in the HPLC database were characterized by exoglycosidase treatments, desulfation reactions, and mass spectrometric techniques.

**REFERENCES**

Bartels MF, Winterhalter PR, Yu J, Liu Y, Lommel M, Mohrlen F, Hu H, Feizi T, Westerlind U, Ruppert T, Strahl S (2016) Protein O-Mannosylation in the Murine Brain: Occurrence of Mono-O-Mannosyl Glycans and Identification of New Substrates. PLoS One 11: e0166119. https://doi.org/10.1371/journal.pone.0166119

Nakagawa H, Kawamura Y, Kato K, Shimada I, Arata Y, Takahashi N (1995) Identification of neutral and sialyl N-linked oligosaccharide structures from human serum glycoproteins using three kinds of high-performance liquid chromatography. Anal Biochem 226: 130-8

Nandi S, Cioce M, Yeung YG, Nieves E, Tesfa L, Lin H, Hsu AW, Halenbeck R, Cheng HY, Gokhan S, Mehler MF, Stanley ER (2013) Receptor-type protein-tyrosine phosphatase zeta is a functional receptor for interleukin-34. J Biol Chem 288: 21972-86

Takahashi N, Nakagawa H, Fujikawa K, Kawamura Y, Tomiya N (1995) Three-dimensional elution mapping of pyridylaminated N-linked neutral and sialyl oligosaccharides. Anal Biochem 226: 139-46

Tanabe K, Ikenaka K (2006) In-column removal of hydrazine and N-acetylation of oligosaccharides released by hydrazionolysis. Anal Biochem 348: 324-6

Trinidad JC, Schoepfer R, Burlingame AL, Medzihradszky KF (2013) N- and O-glycosylation in the murine synaptosome. Mol Cell Proteomics 12: 3474-88

Yagi H, Takahashi N, Yamaguchi Y, Kimura N, Uchimura K, Kannagi R, Kato K (2005) Development of structural analysis of sulfated N-glycans by multidimensional high performance liquid chromatography mapping methods. Glycobiology 15: 1051-60
